# The significance of the oxidative stress markers in the one-year prognosis of patients with acute ischemic stroke: a case-control study

**DOI:** 10.1186/s12883-021-02257-x

**Published:** 2021-07-02

**Authors:** Sheida Shaafi, Fina Hadisi, Mahsa Mahmoudinezhad, Hamidreza Razmi, Seyed Aria Nejadghaderi, Mohammad Khalili

**Affiliations:** 1 Department of Neurology, School of Medicine, Tabriz University of Medical Sciences, Tabriz, Iran; 2 Neurosciences Research Center, Tabriz University of Medical Sciences, Tabriz, Iran; 3 Student Research Committee, Department of Community Nutrition, Faculty of Nutrition and Food Science, Tabriz University of Medical Sciences, Tabriz, Iran; 4 Student Research Committee, Department of Nutrition, Faculty of Nutrition and Food Science, Tabriz University of Medical Sciences, Tabriz, Iran; 5 School of Medicine, Shahid Beheshti, University of Medical Sciences, Tehran, Iran; 6 Cancer Immunology Project (CIP), Universal Scientific Education and Research Network (USERN), Tehran, Iran; 7 Multiple Sclerosis Research Center, Neuroscience institute, Tehran University of Medical Sciences, Tehran, Iran

**Keywords:** Stroke, Total antioxidant capacity, Malondialdehyde, Modified Rankin scale, National Institutes of Health stroke scale, Case-control study

## Abstract

**Background:**

Stroke is a major cause of mortality and morbidity. Also, free radicals and oxidative stress are deleterious factor in the stroke progression. We aimed to evaluate the association between oxidative stress markers and odds of having risk factor for stroke or developing stroke.

**Methods:**

The present case-control study was conducted on 556 participants in Imam-Reza hospital, Tabriz, Iran. Subjects were divided into three group, including individuals with acute ischemic stroke, those who were at risk of stroke, and healthy controls. All enrolled participants except for controls underwent neurological examinations and brain magnetic resonance imaging (MRI). Stroke-related disability and stroke severity were evaluated by modified Rankin Scale (mRS) and National Institutes of Health Stroke Scale (NIHSS), respectively. Serum malondialdehyde (MDA) level and total antioxidant capacity (TAC) were measured within 48 h of the initiation of stroke. One-way ANOVA and Chi-square tests were used for comparing characteristics between groups. Multivariable logistic regression was implemented for odds of stroke based on MDA and TAC quartiles. Also, Spearman’s correlation was utilized.

**Results:**

Serum MDA, systolic and diastolic blood pressure, cholesterol, and triglyceride were significantly higher in the stroke group than controls. High levels of MDA were associated with increased development of stroke (*P*-value < 0.001), however TAC and MDA were not associated with having risk factors for stroke (*P*-value = 1.00 and 0.27, respectively). Also, TAC level was negatively associated with baseline (*ρ* = − 0.28; *P*-value = 0.04) and follow-up (*ρ* = − 0.31; *P*-value = 0.03) NIHSS scores. Moreover, MDA was correlated with mRS score at follow-up (*ρ* = − 0.26; *P*-value = 0.04).

**Conclusions:**

The balance between antioxidants and oxidants markers might reveal a new approach in this context. Further studies are warranted to identify the source of oxidative stress as well as cessation of the production of oxygen radicals in stroke.

## Background

According to the findings of the Global Burden of Disease (GBD) study, the disability-adjusted life-years (DALYs) attributable to stroke were 116.4 million (95% uncertainty interval (UI): 111.4, 121.4 million) in 2016 globally, in which hemorrhagic stroke had a higher proportion than the ischemic type [1]. Almost 80.1 million prevalent cases of stroke were estimated in 2016 worldwide [1]. Over 1990–2016, the age-standardized incidence and mortality rate of stroke declined by 36.2 and 8.1%, respectively. However, it is the second cause of leading death in the world after cardiovascular diseases [2–4]. The incidence of stroke initiated to continuously increase form the age of 30 and it is not significantly higher in men [1].

Oxidative stress is defined as an imbalance between pro- and anti-oxidants, which has been implicated in the pathogenesis of several chronic diseases such as stroke [5]. It plays an important role in the central nervous system and can directly cause tissues damage through several mechanisms [6]. The brain uses glucose as its source of energy, and it requires a steady flow of blood and glucose due to low capacity of energy storage in the brain [7]. The low blood flow decreases the amount of oxygen and glucose, which follows a cascade of events that leads to production of reactive oxygen species (ROSs) and free oxygen radicals [7, 8]. ROSs are necessary for various functions such as a vascular tunic, oxygen pressure monitoring, and erythropoietin production in low concentrations. In contrast, excessive amounts of oxidants may irreversibly oxidize macromolecules and cause severe cell injury [9]. Antioxidant defense system is a special mechanism of dealing with damages induced by free radicals in the body. Healthy individuals have a balance between the production of free radicals and antioxidant defense system, but a dysregulation in this balance induces the oxidative stress production that contributes to progression of stroke [10, 11].

The oxidative stress can be measured using the oxidized products of macromolecules such as nucleic acids, lipids, proteins, and deoxyribonucleic acid (DNA). Lipid peroxides are unstable lipid radicals, which are derived from the oxidation of polyunsaturated fatty acids and can be converted to a different composition such as malondialdehyde (MDA) [12]. MDA can cause irreversible disruption of the enzymes, receptors, and membrane transfer mechanisms [13]. In addition, it has been shown a direct correlation between increases in MDA and poor functional recovery in acute ischemic stroke [14]. Total antioxidant capacity (TAC) measurement is a useful tool for the evaluation of the antioxidant capacity to prevent oxidative damage to membranes and other cellular components [15].

In the present study, the importance of oxidative stress roles in the pathogenesis of acute ischemic stroke is taken into account. To our best of knowledge, no study has examined the long-term effects of oxidative stress on the clinical outcomes of patients with stroke. The aim of this study was to investigate changing in markers of oxidative stress and antioxidant capacity to find whether there is any correlation between those changes and risk of stroke. Also, the correlation between severity and disability of stroke and biochemical markers were assessed.

## Methods

### Subjects and design

The present case control study was conducted in Imam-Reza hospital in Tabriz, Iran from March 2017 to June 2019. Subsequently, 216 patients with stroke, 152 patients at risk of stroke, and 188 healthy controls were included. Patients with stroke required to have a definite diagnosis of stroke by a physician by using magnetic resonance imaging (MRI) with diffusion-weighted imaging (DWI). Inclusion criteria for the group of at risk population was to have at least one of the underlying diseases, including hypertension, type II diabetes mellitus, and hyperlipidemia. Exclusion criteria were history of hemorrhagic infarction, nervous system diseases, chronic diseases such as chronic kidney, liver, and biliary tract diseases, infectious and autoimmune diseases, antioxidant intake over the past 3 months, and smoking.

Written informed consent was obtained from all of the participants at the beginning of the study. The study protocol was approved by the ethics committee of Tabriz University of Medical Sciences (Ethics number: TBZMED 94/3–4/3). All methods were performed in accordance with the national guidelines and regulations.

### Clinical assessments

An expert neurologist underwent neurological examinations for all enrolled cases, and further evaluation by brain MRI with DWI in order to confirm an acute stroke. Ischemic stroke was defined as focal neurologic deficits due to vascular causes lasting more than 24 h and could not be explained by other causes [16]. Stroke-related disability and stroke severity were evaluated by modified Rankin Scale (mRS) and National Institutes of Health Stroke Scale (NIHSS), respectively [17, 18].

### Biochemical assessments

Blood samples were collected from participants within 48 h following the stroke. Serum MDA level was measured using the thiobarbituric acid reactive substance (TBARS) assay (Radioimmunoassay Kit). TAC was measured by the values extracted from ferric-reducing antioxidant power (FRAP) assay that was adjusted based on Iranian foods. The ability of dietary antioxidants for reducing ferric to ferrous ion was calculated by FRAP and expressed by mmol per 100 g of foods [19]. The overall mean value for similar foods, especially those that exist in Iranian culture such as different kinds of bread were calculated. Multiplication of consumption frequency of each food by their related FRAP values were equal to TAC of each participant in the study.

### Statistical analysis

The included participants were classified into quartiles based on their TAC and MDA. One-way analysis of variance (ANOVA) and Chi-square test were used to compare general and demographic characteristics between the three groups for continuous and categorical variables, respectively. Multivariable logistic regression in two different models, without adjustment and with adjustment for age, sex, and body mass index (BMI), were used in order to evaluate the relationship between TAC and MDA and odds of stroke or having risk factors for stroke. Correlation between all the variables were assessed with the Spearman correlation coefficient. The *P*-values less than 0.05 were considered as statistical significance. The Statistical Package for the Social Sciences (SPSS) version 22.0 (SPSS Inc., Chicago, IL, USA) was used for performing statistical analysis.

## Results

### Baseline characteristics

A total number of 556 participants were included in this study (216 in stroke group; 152 in the group at risk of stroke; and 188 individuals in control group). Overall, the mean age of participants was 72.32 years and 41.01% were females. Moreover, we found a higher mean serum concentration of TAC in the patients at risk of stroke compared to healthy controls and stroke patients (3909.29 μmol/L in at risk group vs. 3905.98 μmol/L in controls and 3687.35 μmol/L in stroke group). Nevertheless, the mean serum MDA level was lower in the at risk of stroke group than the control group (1.75 vs. 1.85 μmol/L) (Table 1). The baseline characteristics of participants in each group are presented in Table 1.

**Table 1:** Baseline characteristics of participants

Variables	Healthy control group (*n* = 188)	Stroke group (*n* = 216)	Stroke risk group (*n* = 152)
Female gender^&^	96 (51.1%)	68 (31.5%)	64 (42.1%)
Age^$^ (year)	73.32 (9.43)	71.76 (9.88)	71.89 (10.22)
BMI^$^ (kg/m^2^)	26.55 (4.93)	26.92 (5.85)	27.03 (6.33)
Length of Hospitalization^$^ (day)	ND	11.06 (20.16)	ND
NIHSS-baseline	ND	8.28 (8.90)	ND
NIHSS-follow-up	ND	5.11 (4.86)	ND
MRS- discharge	ND	2.63 (0.78)	ND
MRS- follow-up	ND	1.78 (1.08)	ND
DBP^$^ (mmHg)	73.62 (9.13)	81.17 (11.92)	87.89 (14.55)
SBP^$^ (mmHg)	123.09 (14.95)	148.08 (28.02)	150.79 (25.93)
Total cholesterol^$^ (mg/dl)	167.17 (42.97)	200.06 (62.03)	176.68 (41.58)
Triglyceride^$^(mg/dl)	119.26 (20.19)	175.20 (91.61)	148.50 (59.29)
LDL^$^ (mg/dl)	97.81 (26.15)	118.80 (62.58)	111.03 (37.81)
HDL^$^ (mg/dl)	42.18 (9.31)	46.10 (12.66)	43.36 (13.09)
Serum TAC^$^ (μmol/L)	3905.98 (879.89)	3687.35 (801.56)	3909.29 (983.87)
Serum MDA^$^ (μmol/L)	1.85 (0.36)	2.08 (0.24)	1.75 (0.41)

Abbreviations: *BMI* Body Mass Index, *DBP* Diastolic Blood Pressure, *NIHSS* National Institutes of Health Stroke Scale, *MRS* Modified Rankin Scale, *SBP* Systolic Blood Pressure, *LDL* Low-Density Lipoprotein, *HDL* High-Density Lipoprotein, *TAC* Total Antioxidant Capacity, *MDA* Malondialdehyde, *ND* Not Determined

^&^ Gender is presented as number (percent); there was no significant differences between gender of three group based on Chi-square test (*P*-value = 0.13)

^$^ These data are presented as mean (standard deviation)

A significant difference was shown among patients with stroke and control group in term of baseline systolic blood pressure (SBP) and diastolic blood pressure (DBP), which were significantly higher in both stroke group and at risk of stroke group than healthy controls. In addition, we found significantly increased levels of triglyceride (TG) and total cholesterol in the stroke group than healthy individuals (*P*-value < 0.001) (Table 2).

**Table 2:** Comparison of baseline characteristics between Healthy control, Stroke and diabetes groups

Variables	Group 1	Group 2	Mean difference	*P*-value*
Age	Healthy control	Stroke group	1.55	0.72
		Stoke risk group	1.42	0.80
BMI	Healthy control	Stroke group	−0.36	0.94
		Stoke risk group	−0.47	0.92
DBP	Healthy control	Stroke group	−7.55	0.00
		Stoke risk group	−14.27	0.00
SBP	Healthy control	Stroke group	−18.71	0.00
		Stoke risk group	−27.70	0.00
Total cholesterol	Healthy control	Stroke group	−32.88	0.00
		Stoke risk group	−9.51	0.69
Triglyceride	Healthy control	Stroke group	−55.94	0.00
		Stoke risk group	−29.24	0.13
LDL	Healthy control	Stroke group	−20.99	0.13
		Stoke risk group	−13.22	0.48
HDL	Healthy control	Stroke group	−3.91	0.34
		Stoke risk group	−1.17	0.91
Serum TAC	Healthy control	Stroke group	218.62	0.46
		Stoke risk group	−3.31	1.00
Serum MDA	Healthy control	Stroke group	−0.23	0.00
		Stoke risk group	0.10	0.35

Abbreviations: *BMI* Body mass index, *DBP* Diastolic blood pressure, *SBP* Systolic blood pressure, *LDL* Low-density lipoprotein, *HDL* High-density lipoprotein, *TAC* Total antioxidant capacity, *MDA* Malondialdehyde

*P-values calculated using one-way ANOVA test

### Predictors of stroke development

No significant association was found between quartiles of serum TAC and odds of stroke neither without adjustment for confounding factors nor after adjustment for them (*P*-value = 0.12 without adjustment; *P*-value = 0.14 after adjustment). However, levels of serum MDA were significantly associated with development of stroke before and after adjustment for confounding factors. Looking at the quartiles of dietary TCA showed that participants in the second quartile had a reduced odds of stroke by 71% (odds ratio (OR) = 0.29; 95% confidence interval (CI): 0.09–0.94). Moreover, increasing the levels of serum MDA was associated with elevated risk of stroke development. In this regard, after adjustment for age, sex, and BMI, the third and fourth quartile had ORs of 7.98 (95% CI: 1.94, 32.80) and 11.97 (95% CI: 2.74, 52.35), respectively (Table 3).

**Table 3:** Odds ratios (ORs) (95% confidence interval) for stroke according to the quartiles of serum total antioxidant capacity (TAC) and malondialdehyde (MDA)

Quartiles of TAC					
Variable	1st quartile	2nd quartile	3rd quartile	4th quartile	*P-trend*^$^
Serum TAC^¥^ (μmol/L)	2939.00	2453.00	4102.00	4916.50	
No. cases/ controls	72/ 32	40/59	44/62	60/35	
Model ^1^	1	0.29 (0.09–0.94)	0.34 (0.11–1.10)	0.66 (0.21–2.11)	0.12
Model ^2^	1	0.37 (0.10–1.25)	0.32 (0.09–1.96)	0.75 (0.23–2.48)	0.14
Quartiles of MDA					
Variable	1st quartile	2nd quartile	3rd quartile	4th quartile	*P-trend*^$^
Serum MDA^¥^ (μmol/L)	1.44	1.91	2.06	2.29	
No. cases/ controls	16/ 60	48/ 72	72/ 32	80/ 24	
Model ^1^	1	2.50 (0.66–9.38)	8.43 (2.11–33.60)	12.50 (2.98–52.30)	0.00
Model ^2^	1	2.12 (0.54–8.31)	7.98 (1.94–32.80)	11.97 (2.74–52.35)	0.00

^¥^ These value is presented as median

^$^ *P-trend* was calculated using logistic regression model by considering the median of each quartile of TAC or MDA as a continuous variable

^1^ Regression model without adjustment

^2^ Regression model adjusted for age, gender, and body mass index (BMI)

### Predictors of having risk factors for stroke

No significant association was found between serum levels of TAC and MDA with having risk factors for stroke. Lower levels of MDA were associated with reduced risk for developing stroke risk factors. However, fourth quartile level of serum TAC had lower risk for having stroke risk factors than the third quartile (OR = 0.50 (95% CI: 0.14, 1.79) vs. 0.80 (95% CI: 0.22, 2.93) in the adjusted model). The second quartile of MDA levels had almost the most significant association with not having risk factors for stroke compared to other quartiles of MDA and TCA levels (OR = 0.32 (95% CI: 0.10–1.01)) (Table 4).

**Table 4:** Odds ratios (ORs) (95% confidence interval) for having risk factors of stroke according to the quartiles of serum total antioxidant capacity (TAC) and malondialdehyde (MDA)

Quartiles of TAC					
Variable	1st quartile	2nd quartile	3rd quartile	4th quartile	*P-trend*^$^
Serum TAC^¥^ (μmol/L)	2939.00	2453.00	4102.00	4916.50	
No. cases/ controls	40/ 32	36/ 60	40/ 40	36/ 64	
Model ^1^	1	0.4 (0.13–1.66)	0.80 (0.22–2.87)	0.51 (0.14–1.79)	0.98
Model ^2^	1	0.49 (0.14–1.76)	0.80 (0.22–2.93)	0.50 (0.14–1.79)	1.00
Quartiles of MDA					
Variable	1st quartile	2nd quartile	3rd quartile	4th quartile	*P-trend*^$^
Serum MDA^¥^ (μmol/L)	1.44	1.91	2.06	2.29	
No. cases/ controls	68/ 60	28/ 72	32/ 32	24/ 24	
Model ^1^	1	0.34 (0.11–1.04)	0.88 (0.26–2.93)	0.88 (0.23–3.32)	0.30
Model ^2^	1	0.32 (0.10–1.01)	0.92 (0.27–3.12)	1.03 (0.26–4.06)	0.27

^¥^ These value is presented as median

^$^ *P-trend* was calculated using logistic regression model by considering the median of each quartile of TAC or MDA as a continuous variable

^1^ Regression model without adjustment

^2^ Regression model adjusted for age, gender, and body mass index (BMI)

### Correlation between chemical and clinical assessments

We found statistically significant negative correlation between baseline and follow-up levels of NIHSS and TAC (*ρ* = − 0.28 (*P*-value = 0.04) and − 0.31 (*P*-value = 0.03), respectively) and between levels of mRS in follow-up and MDA (*ρ* = − 0.26; *P*-value = 0.04) (Table 5).

**Table 5:** The correlation between TAC or MDA and NIHSS-baseline, NIHSS-follow-up, mRS-discharge, and mRS-follow-up among patients with stroke

	NIHSS-baseline	NIHSS-follow-up	mRS-discharge	mRS-follow-up
TAC	−0.28 (0.04)	−0.31 (0.03)	− 0.12 (0.37)	−0.17 (0.21)
MDA	−0.09 (0.48)	−0.29 (0.07)	− 0.16 (0.23)	−0.26 (0.04)

Values are presented as Spearman’s rho (*P*-value).

Abbreviations: *TAC* Total antioxidant capacity, *MDA* Malondialdehyde, *NIHSS* National Institutes of Health Stroke Scale, *mRS* Modified Rankin Scale

## Discussion

Findings of this hospital-based case-control study demonstrated that TAC levels despite MDA levels, were lower in patients with stroke. Also, it showed that MDA level is a better predictor of stroke development than TCA, while none of these measures was significantly associated with having risk factors for stroke. Furthermore, we found a negative correlation between clinical tools (i.e. NIHSS and mRS) and chemical measures (i.e. TAC and MDA).

Numerous studies have investigated the TAC and MDA levels in stroke patients and showed that serum TAC levels in stroke cases were significantly lower [20, 21] and MDA levels were higher than the control group [22, 23]. A case-control study on 195 hospitalized cases with stroke and 195 healthy controls in Iranian populations which were categorized into three groups showed that the top tertile of dietary TAC had lower chance to have stroke than the bottom tertile (OR = 0.49; 95% CI: 0.23, 1.00), although our study revealed a significant protective association between the bottom quartile of TCA and stroke (OR = 0.29; 95% CI: 0.09, 0.94) [24]. The discrepancy might be due to different methods for determining categories. Moreover*, the article by* Guldiken et al. which categorized participants into diabetic stroke, nom-diabetic stroke, and healthy controls showed that TAC levels were significantly higher in diabetic acute stroke patients than in non-diabetic ones (10.03 vs. 5.97 mM; *P*-value < 0.001) and was higher in diabetic patients with stroke compared to the control group (10.03 vs. 5.44 mM; *P*-value < 0.001) [25]. Opara et al. found that the total antioxidant capacity was depleted in diabetic patients compared to normal subjects [26]. On the contrary, Savu et al. showed that the TAC of plasma, despite of high oxidative stress levels, was increased in patients with uncomplicated type II diabetes [27]. In the present study, the TAC levels of the patients at risk of stroke did not show any significant differences from the control group, whereas it was higher than the patients with stroke and healthy controls. It might be due to the *different assays* for determination of TAC.

*The study by* Al-Rawi et al. was conducted on *50 patients* with ischemic stroke, 75 participants with a risk factor for stroke, including diabetes, hypertension, and ischemic heart disease, and 25 healthy *individuals*. MDA levels were measured in the serum and saliva of subjects and showed that MDA levels in both groups were significantly higher than the control group (*P*-value < 0.001) [28]. Our findings confirmed that MDA has a significant increasing association with stroke occurrence, while this association was not significant in patients who were at risk of stroke. We propose that significant increase in MDA level in stroke is a reflection of increased MDA production and oxidative stress in cerebral ischemia since the top quartile of MDA was in higher risk of stroke compared to the bottom quartile, although they were not significant.

A case-control study on 50 patients with stroke and 50 healthy controls represented higher levels of MDA in cases than controls (3.31 vs. 1.62 nmol/ml; *P*-value < 0.0001) [29]. In this regard, the article by Bir et al. showed significantly greater MDA values in both atherothrombotic ischemic stroke and with lacunar infarction compared to healthy controls (*P*-value < 0.001) [30]. It has been suggested that blood or neural lipids may be the source of lipid peroxidation caused by ischemia. In addition, increased cytosolic calcium leads to the activation of phospholipases and proteases during ischemia and can lead to conversion of xanthine dehydrogenase to xanthine oxidase or activation of protein kinase. Consequently, these activated enzymes can be the cause of the increased free radicals [31].

Our study also compared the correlation between TAC or MDA with NIHSS-baseline, NIHSS-follow-up, mRS-discharge, and mRS-follow-up. A cohort study on 42 patients with acute ischemic stroke found no significant association between severity of stroke based on baseline NIHSS and level of MDA (*P*-value = 0.60), whereas there was a significant positive correlation between level of MDA and mRS after 3 months of follow-up (*r* = 0.54; *P*-value = 0.001) [32]. In addition, Yaseen et al. revealed that level of MDA on the 7th day had a positive correlation with NIHSS and mRS scores at that day (*r* = 0.335; *P*-value = 0.024 for NIHSS and *r* = 0.342; *P*-value = 0.022) [33]. We found a negative correlation between levels of MDA and TAC, and NIHSS and mRS. Our findings are in accordance with a study on 34 ischemic stroke patients and 34 healthy controls that showed a negative correlation between total antioxidant status (TAS) and NIHSS values, even though it was not significant (*r* = − 0.17; *P*-value = 0.34) [34]. Moreover, another study on acute ischemic stroke patients and healthy controls showed that TAC levels were negatively correlated with NIHSS scores (*r* = − 0.38; *P*-value = 0.02) [21]. The differences in results of our study with mentioned articles can be due to differences in methods of measurement of factors, especially oxidative stress parameters, time to assess the values, and study participants.

The strength of this study is that it is among pioneer studies which included a group of participants who were potentially at risk of having stroke, while *several previous studies* compared serum levels of oxidative markers only between stroke cases and healthy controls. However, our study had some limitations. First, we adjusted multiple logistic regression test by age, sex, and BMI, while other potential confounding and risk factors, especially atrial fibrillation for stroke were not included in our analysis [35]. Second, selection and recall bias could have influenced the results because of susceptibility of case-control studies. Third, we could not reach to a cause-effect relationship because of the observational design of this study. Fourth, body composition might have effects on inflammatory factors [36], while the study did not include data on some body composition components measures such as fat mass.

## Conclusions

In the light of present findings, it seems that MDA is a better predictor of stroke than TCA, while both TCA and MDA might not be recommended to use for prediction of having stroke risk factors. In addition, TCA and MDA had negative association with severity and disability of stroke. Altogether, it will be worthwhile to pursue oxidative stress role in stroke pathogenesis and it is needed to be designed further large scale studies to investigate the roles of TAC and MDA in patients with stroke.

## Data Availability

The datasets generated and/or analyzed during the current study are not publicly available due for they are personal data but are available from the corresponding author on reasonable request.

## Abbreviations

GBD: Global Burden of Disease
DALY: Disability-adjusted life-year
UI: Uncertainty interval
ROS: Reactive oxygen species
DNA: Deoxyribonucleic acid
MDA: Malondialdehyde
TAC: Total antioxidant capacity
MRI: Magnetic resonance imaging
DWI: Diffusion-weighted imaging
mRS: Modified Rankin Scale
NIHSS: National Institutes of Health Stroke Scale
TBARS: Thiobarbituric acid reactive substance
FRAP: Ferric-reducing antioxidant power
ANOVA: Analysis of variance
BMI: Body mass index
SBP: Systolic blood pressure
DBP: Diastolic blood pressure
TG: Triglyceride
OR: Odds ratio
CI: Confidence interval
